# Responses of vegetation cover to hydro-climatic variations in Bosten Lake Watershed, NW China

**DOI:** 10.3389/fpls.2024.1323445

**Published:** 2024-04-16

**Authors:** Xiangyu Ge, Jianli Ding, Nigenare Amantai, Ju Xiong, Jingzhe Wang

**Affiliations:** ^1^ College of Geography and Remote Sensing Sciences, Xinjiang University, Urumqi, China; ^2^ Xinjiang Key Laboratory of Oasis Ecology, Xinjiang University, Urumqi, China; ^3^ Key Laboratory of Smart City and Environment Modelling of Higher Education Institute, Xinjiang University, Urumqi, China; ^4^ Institute of Ecology, College of Urban and Environmental Sciences, Key Laboratory for Earth Surface Processes of the Ministry of Education, Peking University, Beijing, China; ^5^ Institute of Applied Artificial Intelligence of the Guangdong-Hong Kong-Macao Greater Bay Area, Shenzhen Polytechnic University, Shenzhen, China; ^6^ School of Artificial Intelligence, Shenzhen Polytechnic University, Shenzhen, China

**Keywords:** surface water, NDVI, spatiotemporal variations, time-lag effect, EEMD, Bosten Lake

## Abstract

Amidst the backdrop of global climate change, it is imperative to comprehend the intricate connections among surface water, vegetation, and climatic shifts within watersheds, especially in fragile, arid ecosystems. However, these relationships across various timescales remain unclear. We employed the Ensemble Empirical Mode Decomposition (EEMD) method to analyze the multifaceted dynamics of surface water and vegetation in the Bosten Lake Watershed across multiple temporal scales. This analysis has shed light on how these elements interact with climate change, revealing significant insights. From March to October, approximately 14.9–16.8% of the areas with permanent water were susceptible to receding and drying up. Both the annual and monthly values of Bosten Lake’s level and area exhibited a trend of initial decline followed by an increase, reaching their lowest point in 2013 (1,045.0 m and 906.6 km2, respectively). Approximately 7.7% of vegetated areas showed a significant increase in the Normalized Difference Vegetation Index (NDVI). NDVI volatility was observed in 23.4% of vegetated areas, primarily concentrated in the southern part of the study area and near Lake Bosten. Regarding the annual components (6 < T < 24 months), temperature, 3-month cumulative NDVI, and 3-month-leading precipitation exhibited the strongest correlation with changes in water level and surface area. For the interannual components (T≥ 24 months), NDVI, 3-month cumulative precipitation, and 3-month-leading temperature displayed the most robust correlation with alterations in water level and surface area. In both components, NDVI had a negative impact on Bosten Lake’s water level and surface area, while temperature and precipitation exerted positive effects. Through comparative analysis, this study reveals the importance of temporal periodicity in developing adaptive strategies for achieving Sustainable Development Goals in dryland watersheds. This study introduces a robust methodology for dissecting trends within scale components of lake level and surface area and links these trends to climate variations and NDVI changes across different temporal scales. The inherent correlations uncovered in this research can serve as valuable guidance for future investigations into surface water dynamics in arid regions.

## 1 Introduction

Climate change is causing widespread impacts on both the environment and human society. The latest IPCC Sixth Assessment Report (AR6) has underscored the alarming fact that global average surface temperatures from 2011 to 2020 were 1.1°C higher compared to 1850–1900 (Kikstra et al., 2022), with an unprecedented warming rate for at least two millennia (Hantemirov et al., 2022). The Nationally Determined Contributions (NDC) of 2021 warn that if greenhouse gas emissions persist, we could witness more than a 1.5°C increase in global temperatures by 2030 (Fu et al., 2022; Saisirirat et al., 2022). This escalation of global warming poses an escalating threat, especially for ecosystems in arid and semiarid regions, notably lakes and vegetation systems (Rustad, 2008; Wu et al., 2021; Hao and Li, 2022; Wang et al., 2022).

Lakes, being exceptionally sensitive to natural fluctuations, serve as invaluable indicators of global climate change and local environmental shifts (Adrian et al., 2009; Zhang et al., 2018b; Wang et al., 2019a). In arid and semi-arid regions, water plays a pivotal role in shaping ecosystems, directly influencing the development and evolution of aquatic ecosystems and their counterparts (Ndehedehe et al., 2018; Zhang et al., 2018a; Gao et al., 2023). Inland lakes, integral to regional water resources, significantly impact the eco-environmental well-being of oasis areas, particularly for vegetation ecosystems (Liu et al., 2018b; Xue et al., 2019; Zhang et al., 2022b). Vegetation, acting as a vital nexus between soil, atmosphere, and water, drives material circulation and energy exchange in various ecosystems (Sylvain and Wall, 2011; Wang et al., 2023). Vegetation is often used as an indicator of ecological conditions. Thus, exploring the dynamic fluctuations of lakes and vegetation cover in arid regions and understanding how they respond to hydroclimatic variations are imperative for regional ecological conservation and the pursuit of sustainable development goals (SDGs).

In arid regions, lake water resources (e.g., surface area, water level, and volume), vegetation (e.g., Normalized Difference Vegetation Index (NDVI), net primary productivity, and gross primary productivity), and hydroclimatic factors (e.g., temperature, precipitation, and runoff) have garnered global attention (Jiapaer et al., 2011; Voss et al., 2013; Chen et al., 2020; Kooch et al., 2022; Liu et al., 2023). The relationships between lake surface area and hydroclimatic factors and between NDVI and hydroclimatic factors have been extensively scrutinized across various spatial scales (Shen et al., 2017; Nourani et al., 2021; Ogou et al., 2022; Gbetkom et al., 2023). Previous studies have illustrated the strong correlations between lake surface area and variables like temperature and precipitation in arid regions. Additionally, researchers have delved into the mechanisms governing vegetation responses to changing environments (Zhao et al., 2020; Han et al., 2021). However, vegetation dynamics in arid regions are not solely influenced by regional climate; they are also closely tied to water conditions, especially pertaining to lake water resources (Yang et al., 2020; Tuersun et al., 2020; Hou and Rusuli, 2022). This underscores the potential for complex feedback loops linking vegetation, hydroclimatic factors, and lake surface area. Consequently, it is insufficient to study one-to-one relationships in isolation. In the northwestern inland region of China, the regional climatic pattern has shifted from warm-dry to warm-wet, particularly since the 21st century (Fahu et al., 2023). Consequently, both regional lake surface areas (and water levels) and NDVI have exhibited notable transformations, characterized by both definite patterns and occasional anomalies (Ge et al., 2022b). Bosten Lake, situated in China, is the biggest freshwater lake in arid regions (Wang et al., 2018). Yet, the interplay among lake surface area, NDVI, and hydroclimatic factors within the Bosen Lake watershed remains enigmatic. In the upper reaches of this watershed, the Bayanbulak Grassland is the source of many rivers, such as the Kaidu, Ili, and Manas rivers, serving as a crucial ecological barrier in arid Xinjiang (Ye et al., 2017; Yao et al., 2018; Cheng et al., 2023). Meanwhile, the Kongque River in the lower reaches is not only the primary contributor of water to the Tarim River but also a significant contributor to the Lop Nur lake, playing a pivotal role in maintaining the ecological equilibrium of the entire Tarim watershed (Chen et al., 2006; Yang et al., 2020; Zhang et al., 2021). Although regional annual hydrological and meteorological factors, lake area, and vegetation cover in the Bosen Lake watershed have been studied to some extent, these studies have primarily focused on the late 20th century. Few attempts have been made to analyze monthly changes in these indicators and their relationships, particularly over the past two decades (Ge et al., 2022a). Given the backdrop of global warming, scrutinizing the intricate relationships among these elements can provide valuable insights into how lake surface area, vegetation, and hydroclimatic factors respond to evolving environments.

While some studies have explored the connection between climate change and water levels, area, and vegetation cover in the Bosten Lake watershed (Maimaiti et al., 2016; Yao et al., 2018; Wufu et al., 2020; Yanfei et al., 2021; Tang et al., 2022). The most have quantified the relative impacts of climate and vegetation variables on a single time scale, often making linear assumptions. This narrow focus overlooks the multi-timescale nature of these interactions, leading to underestimated impacts (Liu et al., 2018a). Consequently, a multi-timescale approach is essential for assessing the relative influence of climate change on vegetation and surface water changes. The ensemble empirical mode decomposition (EEMD) is particularly adept at handling non-stationary signals (Wu and Huang, 2009), This method is particularly effective in handling nonstationary signals. The residue captures the nonlinear trend of the data, including trend reversals, in addition to monotonic trends (Ji et al., 2014; Pan et al., 2018). Compared to wavelet analysis, EEMD is less affected by the length of the time series and is more adaptable to inherent data characteristics. It also reduces mode mixing and improves noise handling. EEMD is a reliable tool for extracting underlying physical information from nonlinear and nonstationary time series (Pan et al., 2018). Thus, EEMD stands as a suitable technique for uncovering the relationships between multi-time scale climate, surface water, and vegetation, and has been widely used (Wen et al., 2017; Liu et al., 2018a, 2021).

Given the challenges that have arisen in the Bosten Lake watershed over the past two decades, characterizing the regional lake surface area, vegetation, and hydroclimatic factors is paramount. In this study, we employed the EEMD method to extract vegetation dynamics, surface water variations, and climate changes at multiple timescales. We then explored the relationships between these variables across various timescales, enriching our scientific understanding of conservation and restoration efforts for fragile ecosystems dependent on vegetation and water resources. Our objectives are: (1) To identify significant changes in the trends of vegetation, water levels, and area within the Bosten Lake in the past 2 decades. (2) To analyze the trends in the water level, area, NDVI, and climate change at different timescales. (3) To uncover the intricate relationships between these variables at different timescales.

## 2 Materials and methods

### 2.1 Study area

**Figure 1:**
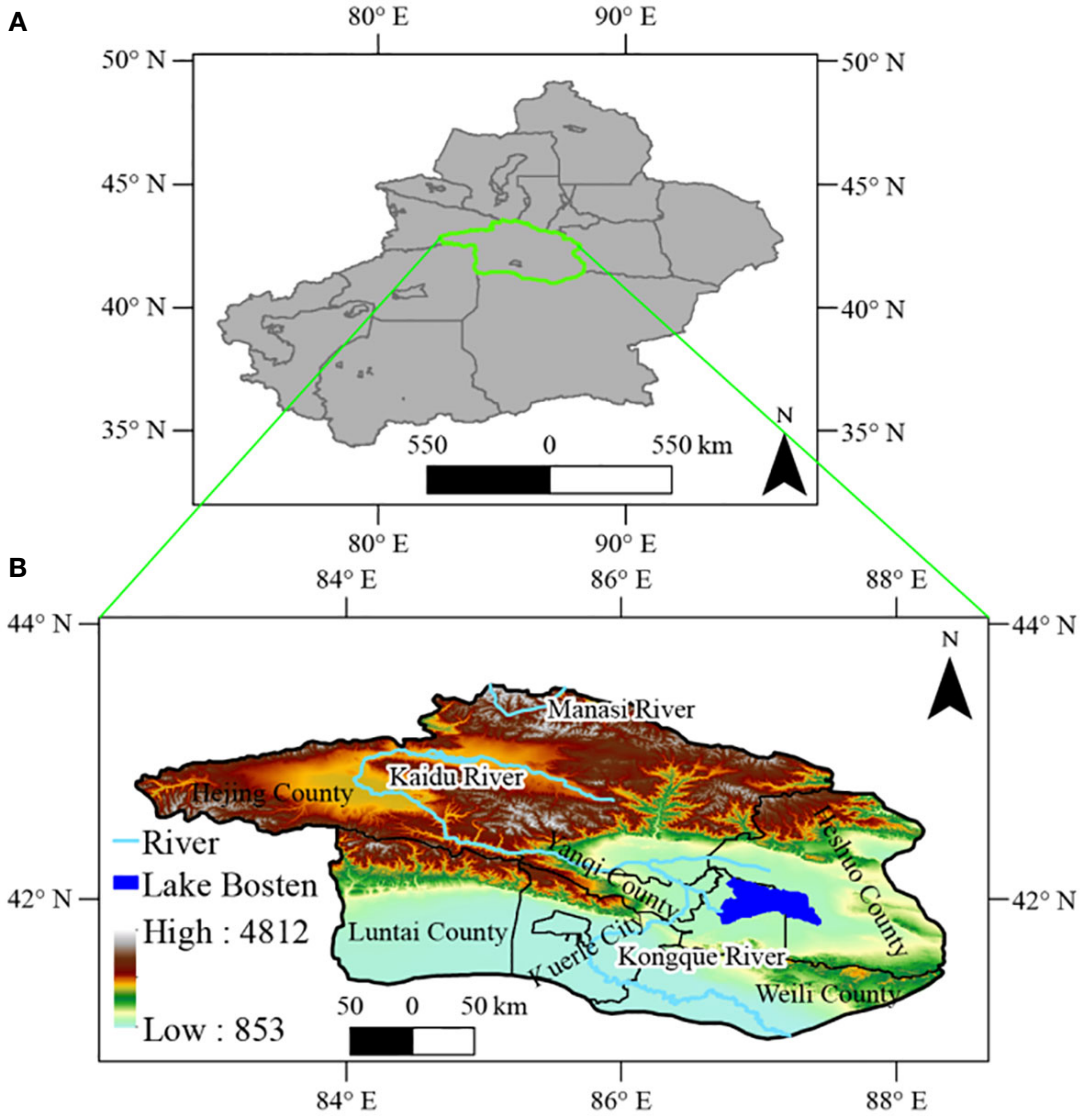
Geographic location **(A)** and DEM map **(B)** of the Bosten Lake Watershed.

The Bosten Lake watershed is situated in the northeastern part of Xinjiang (82°80´–88°63´E, 40°73´–43°57’N), covering approximately 68,687 km^2^ (Tuersun et al., 2020) (**Figure 1**). The watershed has a complex topography consisting of watersheds, mountains, and canyons. It spans an altitude range of 853–4,812 m (Tuersun et al., 2020). Precipitation in this region is mostly concentrated in May-September, with an average annual precipitation of 188.1 mm (Tuersun et al., 2020). Bosten Lake, the largest inland freshwater lake in the watershed, serves both as the tail lake of the Kaidu River and the source of Peacock Lake (Yao et al., 2018). Consequently, fluctuations in water resources directly impact the local ecosystemon development and evolution.

### 2.2 Data acquisition and processing

**Figure 2:**
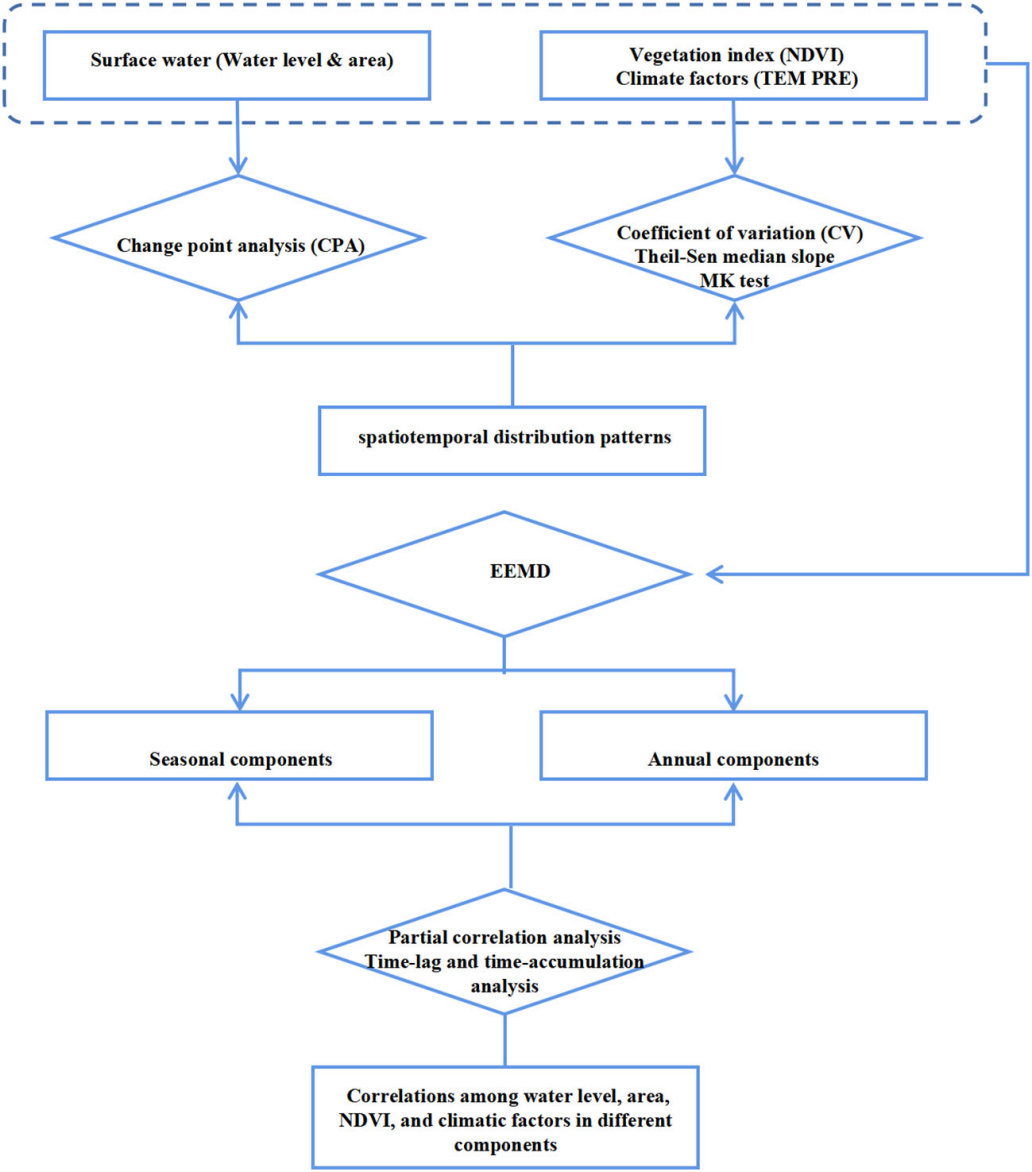
Primary analysis process and framework for this study.

The main steps followed in this study are shown in **Figure 2**.

We sourced the Global Surface Water (GSW) dataset (30 m), provided by the Joint Research Centre (Pekel et al., 2016). This dataset offers maps displaying surface water location and temporal distribution from 1984 to 2021, along with statistics on surface water extent and changes. With an error rates of 1% for misclassification and 5% for omission. Researchers have consistently recognized it as a reliable reference since its 2016 release (Gorelick et al., 2017). We processed this dataset to extract monthly water frequency with a resolution of 1 km, all within GEE platform. Additionally, we employed the Hydroweb project (https://hydroweb.theia-land.fr/) as a database for water-level time series for lakes, reservoirs, and rivers (Crétaux et al., 2011). We collected the monthly water level and area data of the Bosten Lake Watershed from 2002 to 2020.

The Normalized Difference Vegetation Index (NDVI), derived from remote sensing, gauges vegetation’s capacity to absorb solar radiation and reflects its coverage and growth to a certain extent. Consequently, NDVI is a key metric for monitoring ecological and environmental changes on regional and global scales. Monthly NDVI data in the last two decades were obtained from the National Earth System Science Data Center of China (http://www.geodata.cn). This dataset was generated through monthly synthesis, mosaics, and clipping of the MOD13A2 V6 product (Didan, 2015). NDVI values between 0 and 0.1 represent non-vegetated areas (Liu et al., 2018b), so we masked areas with NDVI values below 0.1, considering pixels with NDVI ≥ 0.1 during the growing season (March–October) as stable vegetation areas.

Monthly precipitation and temperature data from 1901 to 2021 (~1 km), were obtained from the National Tibetan Plateau Scientific Data Center (https://data.tpdc.ac.cn/) (Ding and Peng, 2020).

### 2.3 Statistical analyses

In this study, Change point analysis (CPA) was used to estimate the area and hydrology of Lake Bosten via the R package ‘changepoint’ (Killick and Eckley, 2014). We used coefficients of variation to quantify the spatial variability of interannual changes in vegetation (Jiang et al., 2015). heil-Sen median trend analysis and the Mann-Kendall test can be used to analyze pixel-level trends in climatic elements (Fensholt et al., 2012; Deng et al., 2020). We used partial correlation analysis to decouple the interactions between lake level (area), NDVI, precipitation, and temperature. To further explore the relationship between the monthly response of lake level (area) and other variables (NDVI, precipitation, and temperature) (Wang et al., 2013). To further explore response relationships between lake water level (area) and other variables (NDVI, precipitation, and temperature) on a monthly scale, we conducted partial correlation analysis with time-lag (-accumulation), building upon the approach of (Gessner et al., 2013; Amantai et al., 2024).

### 2.4 Ensemble empirical mode decomposition

Natural phenomena time-series data, such as NDVI, climate variables, and hydrological variables, are typically nonlinear and non-stationary (Verma and Dutta, 2013; Wen et al., 2017). Interannual variability is often affected by noise, fluctuations, or mutations, leading to an insufficient understanding of the interannual variability problem (Verbesselt et al., 2010). To analyze the complex interannual changes in climate factors, vegetation changes and hydrological processes, we decomposed these time series using EEMD.

Time series data (t) were decomposed into an intrinsic mode function (IMF) components and a residual using the EEMD method. Each IMF component has a respective mean period T, calculated by counting the number of extrema divided by twice the length of the data (t) (Hawinkel et al., 2015). Following the framework suggested by Hawinkel, the n IMFs and residuals were classified into noise IMFs, annual IMFs, and interannual IMFs based on their mean periods T (as shown in **Figure 2**). Referring to Wen et al. (Wen et al., 2017), in this study, IMFs with T < 6 months were grouped as noise IMFs, those with T between 6 and 24 months were grouped as annual IMFs (seasonal change components), and the remaining IMFs and the residuals were grouped as interannual IMFs, representing overall trend components. The sums of the grouped IMFs were presented as the noise component (Cnoise), annual component (Cannual), and interannual component (Cinterannual) (**Supplementary Figures S1**–**S5**).

## 3 Results

### 3.1 Surface water spatiotemporal distribution pattern

**Figure 3:**
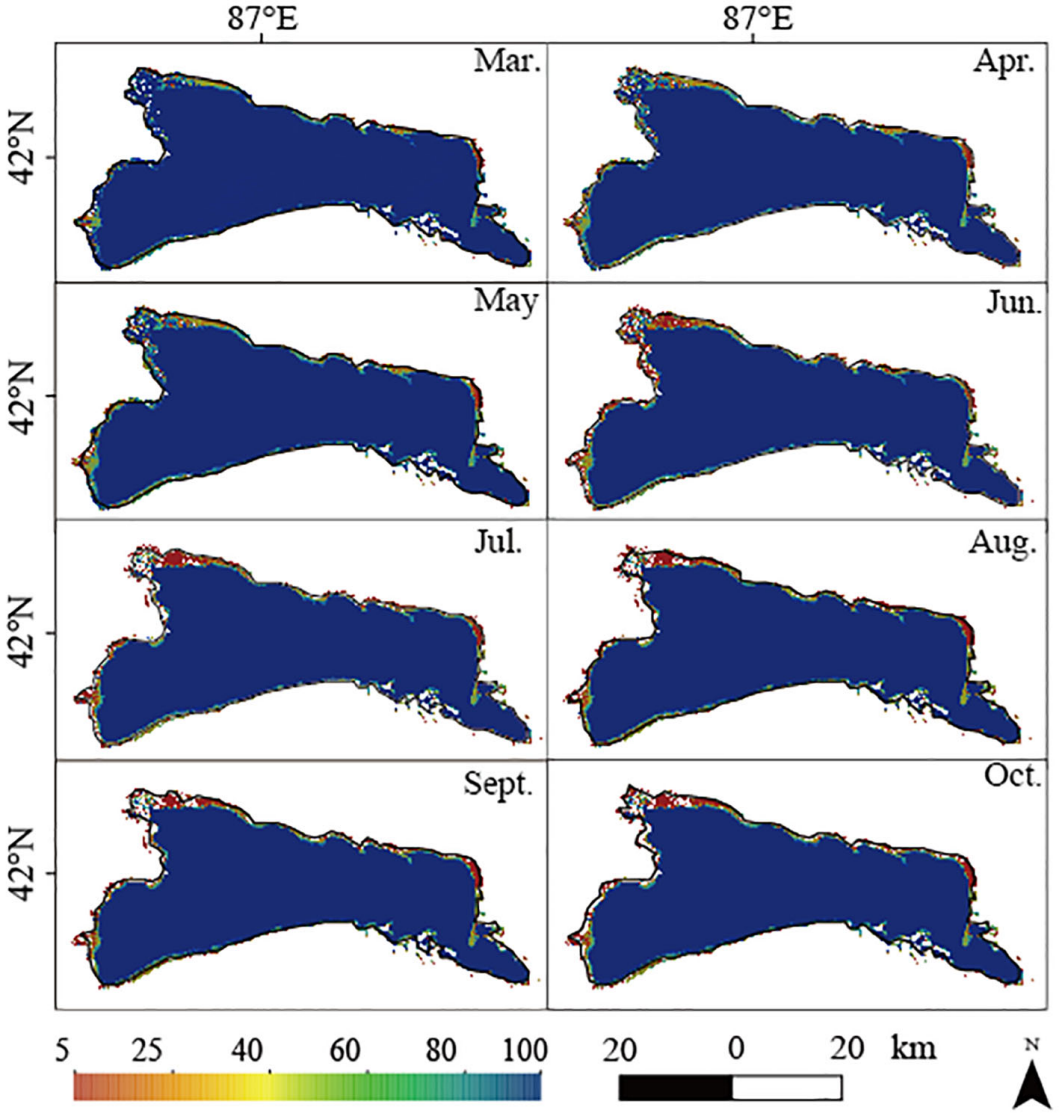
The spatio-temporal variation characteristics of the monthly water frequency.

First, we utilized the GSW data to analyze the monthly water frequency and spatial distribution of each pixel point in Bosten Lake from 2001 to 2020 (excluding data from November to February). The monthly frequency remained constant at 100%, indicating a consistent presence of permanent surface water. However, there were fluctuations. From March to April, the permanent water area increased to approximately 1443.76 km^2^, subsequently declining to its lowest of 1396.40 km^2^ in August, followed by a rebound to 1407.08 km^2^ from September to October. During this period (March to October), approximately lake’s surface area experienced a risk of receding and drying (**Figure 3**).

**Figure 4:**
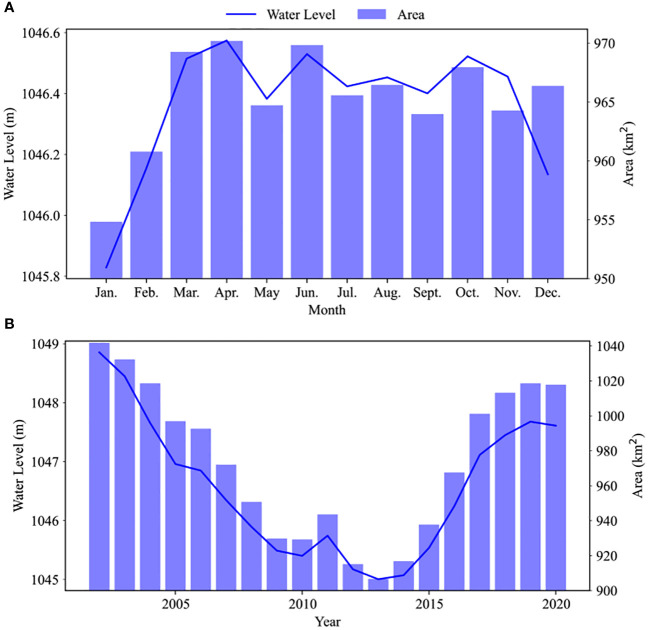
The monthly **(A)** and annual **(B)** average change trends of the area and water level in Bosten Lake.

Next, we examined the monthly and annual average change trends of the area and water level of Bosten Lake (**Figure 4**). The monthly average water level exhibited an upward trend from January to April, stabilizing around 1046.5 m and fluctuating within 0.2 m from April to October, while it decreased from October to December. The lake area peaked at around 966.6 km^2^ from January to April. From April to December, the area varied within a 5 km^2^ range. However, the yearly averages indicated a continuous decline in both water level and area, reaching their lowest points in 2013 (1045.0 m and 906.6 km^2^, respectively) and subsequently increasing to 1047.61 m and 1017.8 km^2^, respectively, in 2020.

**Figure 5:**
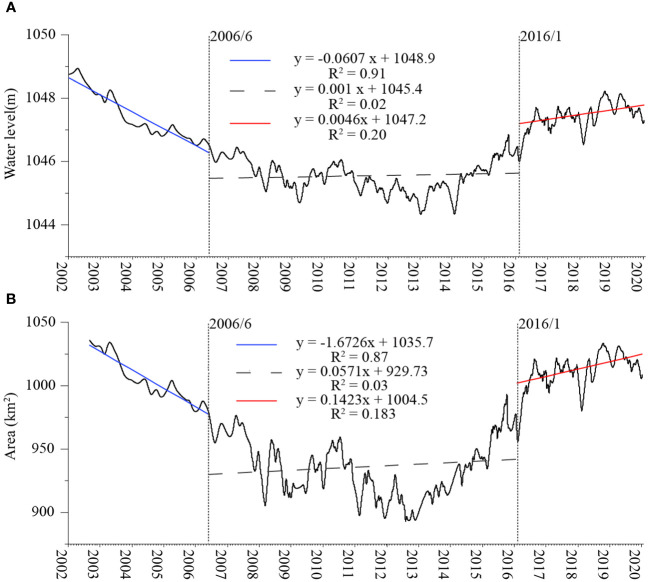
Results of change point analysis of water level **(A)** and area **(B)**.

We applied CPA to explore shifts in the area and water level time series of Bosten Lake using Hydroweb project data (**Figure 5**). Two change points were identified, occurring in June 2006 and January 2016. This analysis revealed three stages of change from 2002 to 2020: a rapid reduction stage (January 2002 to June 2006) with change rates of −0.0607 m/month, and −1.6726 km^2^/month, respectively and an R square as high as 0.91 and 0.87, respectively; a stable period (June 2006 to January 2016) with R squares of only 0.02 and 0.03; a gradual increase stage (January 2016 to December 2020) with increasing rates of 0.0046 m/month and 0.1423 km^2^/month and R squares of 0.2 and 0.18, respectively.

### 3.2 Regional NDVI change characteristics

**Figure 6:**
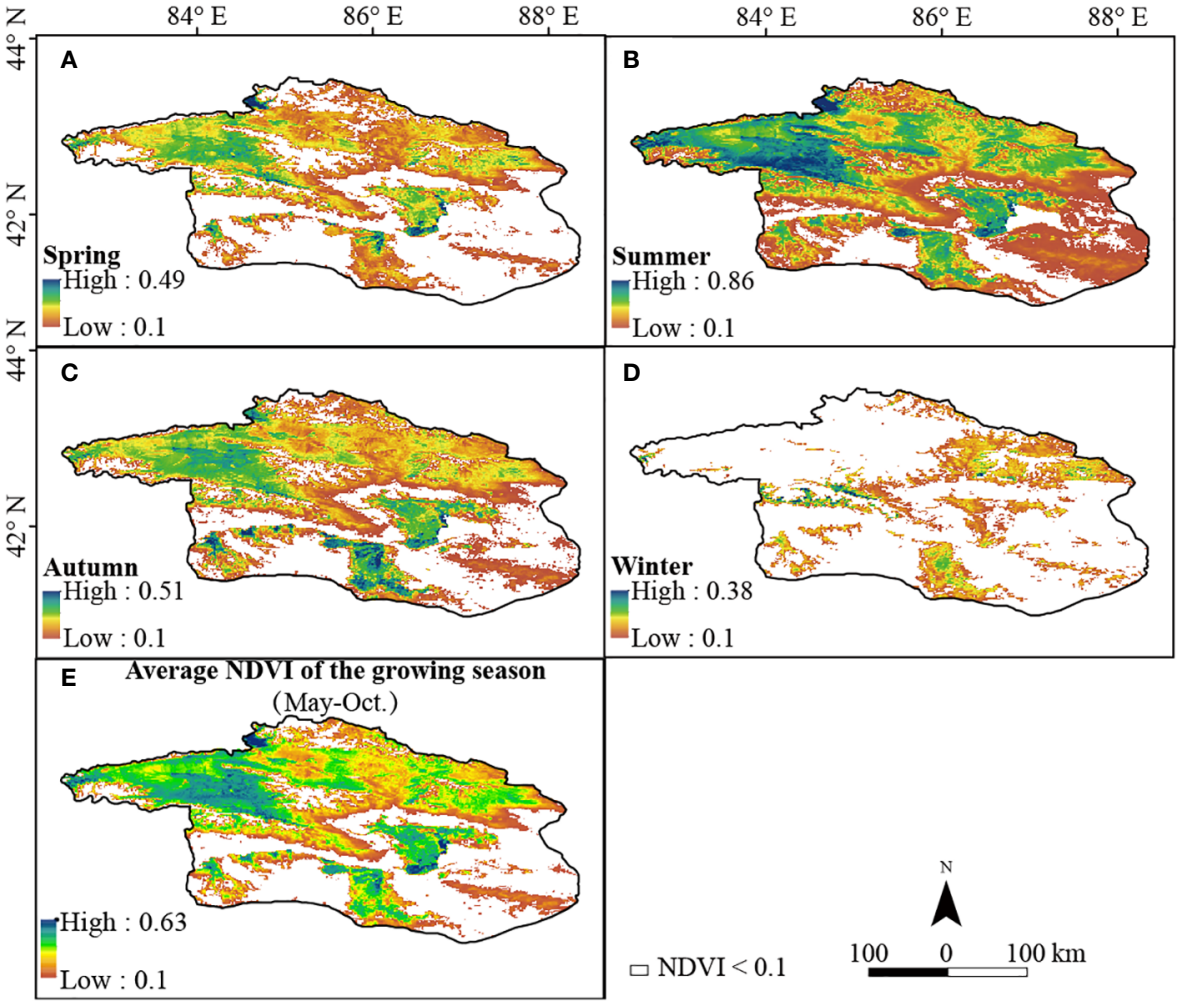
The spatio-temporal variation characteristics of the NDVI in four seasons **(A–D)**. **(E)** is average NDVI of the growing season (May-Oct.).

We analyzed spatio-temporal variations of NDVI (**Figures 6A–D**) in the Bosten Lake Watershed across seasons (i.e., areas with NDVI ≥ 0.1). NDVI increased from spring to winter and then decreased, with mean values of 0.18, 0.34, 0.20, and 0.14, respectively. The average NDVI of the growing season (March to October) (**Figure 6E**) ranged from 0.1 to 0.63 and was 0.27. High NDVI values were mainly concentrated in the upper reaches of the Kaidu River, west of Bosten Lake, and west of the Kongque River from spring to autumn. Conversely, NDVI values decreased in most areas during the winter, except for certain mountainous regions.

**Figure 7:**
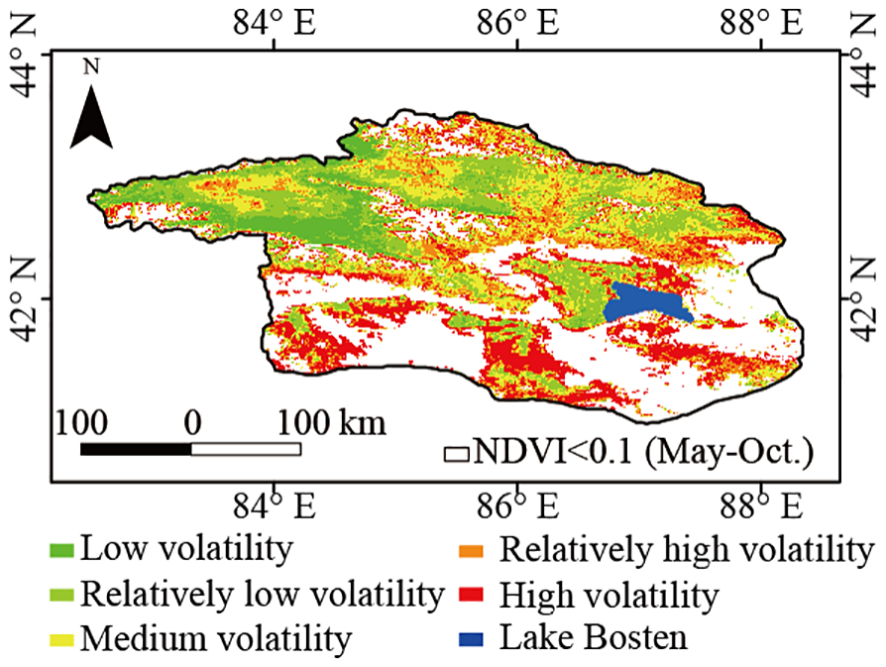
Spatial distribution of the coefficient of variation of the inter-annul NDVI in the Bosten Lake Watershed from 2001 to 2020.

The average coefficient of variation of the NDVI in the past 20 years was 0.17 (**Figure 7**). Lower levels of volatility were predominantly found in the northwestern mountains and upper reaches of the Kaidu River. Conversely, in the plain areas adjacent to the mountain base, specifically in the southern study area and southeast of the Bosten Lake, medium and high levels of volatility were observed. Approximately 66.4% of vegetated areas had medium to high volatility, with 23.4% displaying high volatility (**Table 1**), suggesting significant fluctuations in vegetation coverage within the Bosten Lake watershed.

**Table 1:** Coefficient of variation of NDVI in the Bosten Lake Watershed.

CVNDVI	Volatility degree	Area percentage (%)
≥0.20	High-volatility	23.4%
0.15 ≤ CVNDVI < 0.20	Relative-high volatility	16.9%
0.10 ≤ CVNDVI < 0.15	Medium volatility	26.1%
0.05 ≤ CVNDVI < 0.10	Relatively low volatility	25.1%
<0.05	Low volatility	8.5%

**Figure 8:**
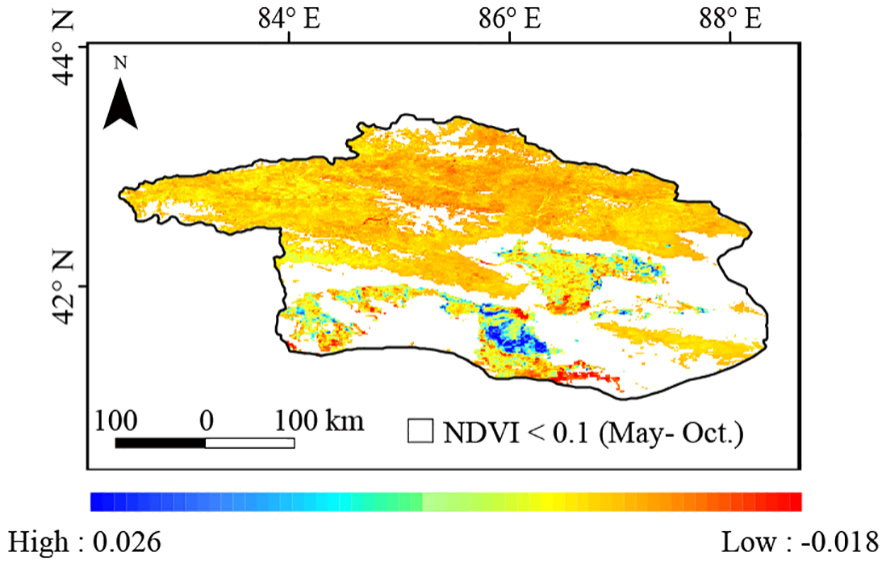
Spatial distribution of the Theil-Sen slopes for NDVI in the Bosten Lake Watershed from 2001 to 2020.

Stable vegetated areas (NDVI ≥ 0.1 during the growing season) exhibited an increase in NDVI from 2001 to 2020 (**Figure 8**), with an average Theil-Sen median slope of 2 (10^-3^·yr^-1^), ranging from −0.018 to 0.026. NDVI improved (i.e., with a Theil-Sen median slope >= 0.0005), remained stable (i.e., with a Theil-Sen median |slope| <= 0.0005), and degraded (i.e., with a Theil-Sen median slope < −0.0005) in 7.7%, 83.3%, and 9.0% of the vegetated areas, respectively. The area with significantly improved NDVI was mainly located west of the Konque River and near Lake Boston, correlating with regions of high volatility.

### 3.3 Spatio-temporal dynamics of precipitation and temperature

**Figure 9:**
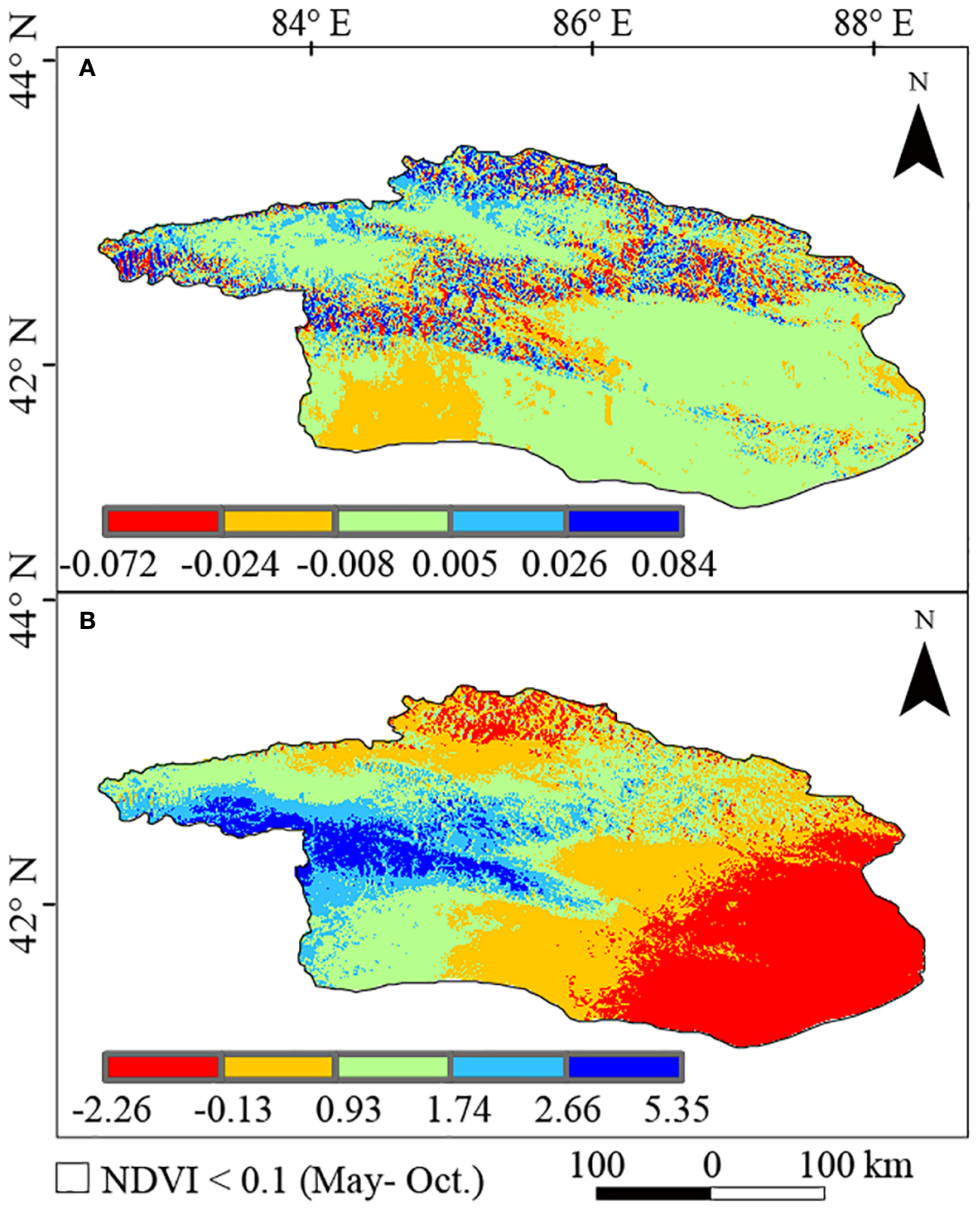
Spatial distribution of the Theil-Sen slopes for temperature **(A)**, precipitation **(B)** in the Bosten Lake Watershed from 2001 to 2020.

Temperature slightly decreased in this study over the last 2 decades (**Figure 9A**), with an average Theil-Sen median slope of −0.002 (°C/yr), ranging from −0.072 to 0.084. Conversely, precipitation increased (**Figure 9B**), with an average Theil-Sen median slope of 0.87 (mm/yr), ranging from −2.26 to 5.35. Temperature and precipitation increased in 34.1% and 88.9% of the study areas, respectively, while they remained stable in 4.3% and 0.1% of the study areas, respectively. However, the temperature decreased in 61.6% of the areas compared to 11.0% for precipitation. Temperature changes were spatially diverse, with some northern areas experiencing increases due to higher terrain, while the lower southern research area and upper reaches of the Kaidu River saw decreases. The changes in precipitation were more uniform in spatial distribution except in the northern and southeastern parts of the study area where there was an increasing trend in precipitation.

### 3.4 Correlations among water level, area, NDVI, and climatic factors in different components

#### 3.4.1 Variation trends of water level, area, NDVI and climate factors in different components

We calculated correlation coefficients among raw values of water level, area, NDVI, and climate variables. Precipitation and temperature showed strong correlations with NDVI (correlation coefficients of 0.82 and 0.90, respectively), but the correlations between water level, area, and other factors did not reach significance (**Supplementary Table S2**).

**Figure 10:**
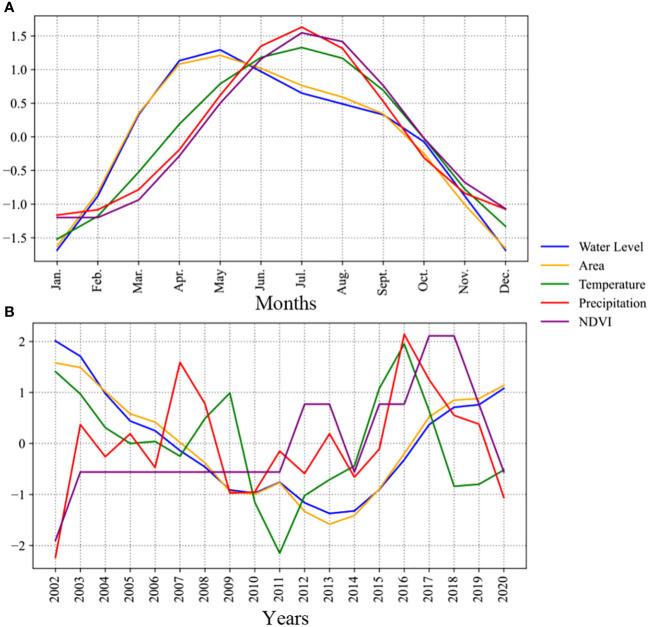
Seasonality **(A)** and trends **(B)** of water level, area, temperature, precipitation and NDVI in the Bosten Lake Watershed.

As water level and area did not exhibit straightforward stationary patterns, we employed the EEMD method to differentiate between their annual and interannual components (**Supplementary Figures S1**–**S5**). By comparing these components across the five parameters, we could validate their changes throughout the observation period (**Figure 10**). Within this plot, each curve’s values are normalized on the sum of amplitudes, combining annual components and averaged seasonal components. Our analysis revealed consistent trends in water level and area throughout the year, reaching their peak in May. In contrast, the variations in NDVI and precipitation displayed synchronization, albeit with a lag compared to the water level and area, peaking in July. Temperature’s seasonal components fell in between water level (area) and NDVI (precipitation) in terms of timing, occurring earlier than precipitation and NDVI, but without the same magnitude of change range as water level (area). Temperature, however, also peaked in July. Over the years, water level and area exhibited a consistent trend of initial decline followed by increase, hitting their lowest point in 2013. Temperature and precipitation showed fluctuating downward and upward trends, respectively, with peaks in 2007, 2009, and 2016, and a trough in 2011 and 2018, while NDVI exhibited an upward trend post-2011.

#### 3.4.2 Inner relationship of water level, area, NDVI, and climate factors in different components

To perform a comprehensive joint analysis of climate, vegetation, and hydrology, we conducted partial correlation, time-lag and time-accumulation analyses of water level (area), NDVI, precipitation, and temperature for the two components. These analyses revealed differences in time-lag and time-accumulated effects between the components when compared with the raw data. In both components, the partial correlation between water level (area) and NDVI, precipitation, and temperature surpassed the significance threshold of 0.05, resulting in significantly improved correlation coefficients. For the annual components, the strongest correlation was observed between temperature and water level (area), reaching up to 0.352 (0.440), indicating a positive relationship. Precipitation followed, exhibiting a correlation coefficient of 0.278 (0.234), while NDVI displayed a negative correlation with water level, with a coefficient of −0.158 (−0.284). In contrast, for the interannual components, the correlation coefficients were generally lower than those in the seasonal components. The values between temperature, precipitation, NDVI, and water level (area) were 0.218 (0.210), 0.147 (0.193), and −0.155 (−0.142), respectively. The time-lag and time-accumulated effects differed as well. In the case of seasonal components, temperature and water level (area) changed in sync, with a 3-month lag between precipitation and water level and a 3-month accumulation effect between NDVI and water level. Conversely, for the annual component, there was a 3-month lag between temperature and water level (area), while precipitation and water level exhibited a 3-month accumulation effect. Notably, NDVI and water level changed simultaneously.

## 4 Discussion

### 4.1 Possible response mechanisms

The energy balance and dynamic processes of the hydrological cycle are at the heart of the complex relationships between vegetation, climate and hydrological parameters. Climate change is an important controlling factor. Naturally, precipitation significantly influences regional water bodies and levels. In arid and semi-arid regions like the Bosten Lake area, lake hydrological conditions are particularly sensitive to inflow runoff. To better understand how water area and level in the study area respond to these influences, we conducted analyses using regional hydrometeorological datasets.

**Figure 11:**
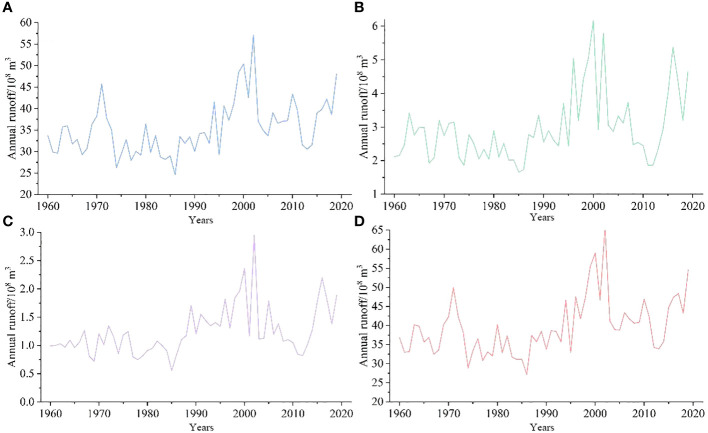
Annual runoff in Bosten Lake Watershed. **(A)** Kaidu River, **(B)** Huangshuigou River, **(C)** Qingshuihe River, and **(D)** Total runoff.

Between 1960 and 2019, the regional average inflow runoff reached 3.97 × 10^9^ m^3^ (**Figure 11**). In terms of the variation trend, the overall inflow increased at a rate of 1.90 × 10^8^ m^3^/10a (*p* < 0.05), but there were also evident phased changes. Overall, the runoff changes in the Kaidu, Huangshuigou, and Qingshui rivers were consistent with the total inflow runoff, with relatively large interannual changes in the Kaidu River. In comparison, the Huangshuigou and Qingshuihe rivers experienced stable changes before the 21st century, and there have been significant interannual fluctuations since then.

**Figure 12:**
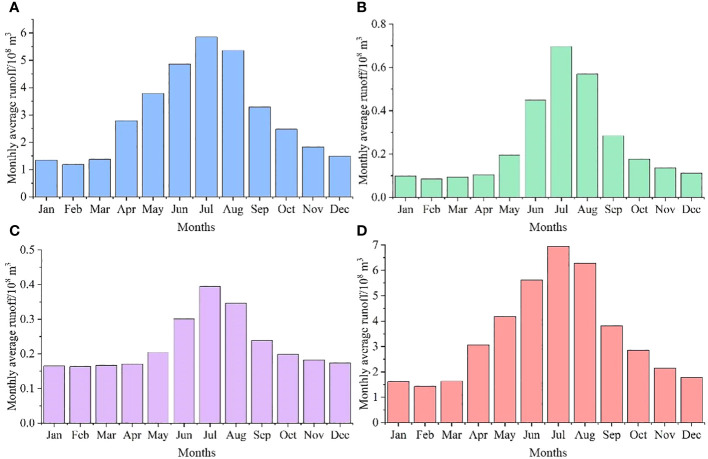
Monthly runoff in Bosten Lake Watershed. **(A)** Kaidu River, **(B)** Huangshuigou River, **(C)** Qingshuihe River, and **(D)** Total runoff.

The average runoff distribution for each month of the Kaidu and Huangshuigou Rivers shows a single-peak pattern. The Kaidu River’s dry season lasts from November to March, accounting for approximately 20% of the year, with the highest runoff occurring from June to August each year, making up approximately 45% of the annual total. The flow occurs in July of each year. Huangshuigou’s dry season lasts even longer, from October to April, accounting for approximately 27% of the annual runoff, while the wet season is mainly from June to August, representing approximately 57% of the annual runoff. As shown in **Figure 12**, the changes in Huangshuigou from October to April of each year are not evident. Beginning in May, the average monthly runoff increased significantly. The runoff distribution is more concentrated from May to September. The runoff from the Qingshuihe River was similar to that from the Huangshuigou River.

Previous research has emphasized that Bosten Lake relies primarily on surface runoff, with the Kaidu River contributing a significant portion (84.7%) compared to precipitation (5.2%) (Yanfei et al., 2021). This suggests that, in this context, inflow, especially from the Kaidu River, has a more substantial impact on the lake’s water level and area, aligning with the correlation between Kaidu River runoff and lake water level and area changes. Notably, fluctuations in runoff from various hydrological stations from 2005 to 2015 did not exhibit an overall trend, coinciding with the relatively stable water level and area of Bosten Lake in 2006 and 2016 (**Figure 5**). Moreover, the stability of the lake water level and area from April to October corresponds to the dry period from November to March for the Kaidu River (**Figure 12**).

The variation in the Kaidu River’s runoff is a complex process influenced by multiple factors. In this watershed, runoff replenishment primarily results from mountain precipitation (61.5%) and snowmelt (38.5%) (Yao et al., 2018). Interestingly, despite precipitation constituting the majority of this influence, the correlation between precipitation and Bosten Lake’s water level and area is not significant.

This phenomenon can be attributed to the distribution of precipitation and snowmelt over the runoff year. Snow accumulation in the Kaidu River watershed begins in November and persists until March (Zhao et al., 2021). With rising temperatures in April and May, snowmelt becomes the primary source of river replenishment (Zhao et al., 2021). During this period, spring snowmelt-induced floods are common, and even on April 24, 2011, they exceeded summer floods (Zhao et al., 2021). Studies have shown that from April to July, snowmelt contributes to over 55% of the annual runoff (Chen et al., 2015). Conversely, precipitation in the Kaidu River watershed over the past 60 years has exhibited a highly uneven trend, primarily concentrated in the summer (Yao et al., 2018). For example, in locations like Huangshuigou, most of the annual precipitation occurs during the summer, with seasonal precipitation contributing up to 90% of the annual total. Monthly variations in annual precipitation can be extreme, reaching up to 135 times (Yao et al., 2018).

Considering these factors, the temporal distribution of precipitation and snowmelt during a wet year, along with their regulatory impacts on aquatic systems, could potentially mask the straightforward correlation between precipitation levels and the fluctuations in both water level and surface area of Bosten Lake. Although precipitation significantly influences overall runoff, in the specific context of Bosten Lake, the relationship between precipitation and lake water level and area might be influenced by intricate factors, rendering it statistically insignificant.

### 4.2 Interference of anthropogenic activities

Human economic activities have profoundly impacted the ecological security and sustainable development of the Bosten Lake watershed over the past decade. Increased human activities, particularly those related to agricultural irrigation, industrial water usage, and domestic water consumption, have influenced the inflow volume of the Bosten Lake (Yao et al., 2018). The expansion of agricultural and irrigation areas, coupled with rapid socioeconomic growth, has led to a rapid increase in water intake and consumption for irrigation, resulting in reduced the inflow volume of the Bosten Lake and subsequent declines in water levels (Yao et al., 2018). The influence of human activities on the lake’s inflow has grown progressively, especially in the 21st century, with their impact intensity reaching 80.80% (Junqiang et al., 2021).

**Figure 13:**
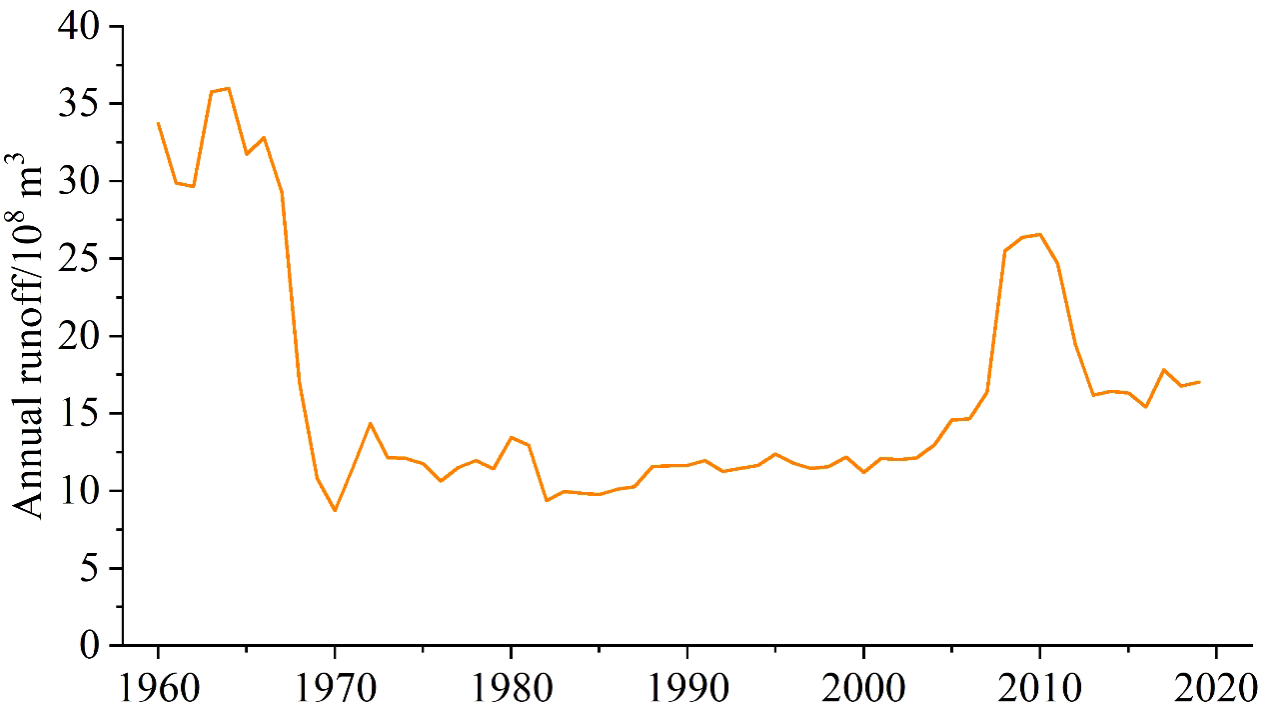
Runoff of Tashidian hydrological station.

Additionally, ecological water transfer projects have affected the Bosten Lake watershed since 2000. While these projects have had positive effects on groundwater recovery and vegetation restoration downstream of the Tarim River (Yao et al., 2018), reduced water inflow from the Kaidu River and increased economic water usage have led to continued declines in Bosten Lake’s water level, leading to severe water scarcity in the downstream Kuche River area and environmental degradation (Junqiang et al., 2021). Fortunately, from 2016 to 2019, ecological water transfer from Bosten Lake to the downstream Kuche River reached a cumulative volume of 1.7 × 10^9^ m^3^, halting the degradation of the downstream watershed (Junqiang et al., 2021). This is confirmed by the changes in flow observed at the Tashidian hydrological station in the Kuche River (**Figure 13**).

In conclusion, human activities have significantly impacted the water resources and ecological environment of the Bosten Lake watershed. Factors such as agricultural irrigation, ecological water transfer, and economic development have variously altered the lake’s hydrological cycle, exacerbated by uncertainties in water resources due to global warming. The future trajectory of Bosten Lake will depend on achieving a balance between human needs and ecological preservation within the framework of sustainable development. While some success has been achieved in ecological restoration, the negative effects of human activities and climate change on wetland water bodies and vegetation must be acknowledged for effective management and control measures. Planned hydrological engineering to regulate seasonal hydrological processes and improve the efficiency of water use may be the key to coping with water resource problems in the future. Addressing the varying influencing factors in different periods and regions is crucial for achieving sustainable development and ecological security in the Bosten Lake wetlands.

### 4.3 Relationship among water level, area, climate, and NDVI variations at multiple time scales

Previous studies examining the correlation between Bosten Lake’s water level and area and climate change have yielded inconsistent conclusions. Dai et al. suggested that annual average evaporation, temperature, and precipitation are closely linked to dynamic changes in the lake’s area (Dai et al., 2020), while Peng et al. found that the interannual variation in the lake area showed no significant correlation with precipitation, evaporation, and accumulated temperature, but exhibited higher correlations with intra-seasonal variations in these meteorological factors (Yanfei et al., 2021). Overall, NDVI in the Bosten Lake watershed was negatively correlated with temperature on an interannual scale, while it was positively correlated with precipitation. On a monthly scale, NDVI exhibited strong positive correlations with both precipitation and temperature. However, limited research exists on the relationship among lake area, water level, and NDVI changes. Our study indicates that, although NDVI maintains a robust positive correlation with precipitation (correlation coefficient of 0.82) and temperature (correlation coefficient of 0.79) on a monthly scale, the correlations between monthly lake water level, area, temperature, precipitation, and NDVI did not reach significance (**Supplementary Table S2**). Nevertheless, through EEMD decomposition, we revealed that the relationship among lake water level, area, climate, and NDVI changed over different time scales, achieving significance (p < 0.05), influenced by the cumulative effects of varying hydrothermal conditions.

At the annual scale (6 < T < 24 months), the strongest positive correlation was observed among the lake water level, area, and temperature, exhibiting simultaneous changes (**Supplementary Table S3**). This correlation is likely due to rising temperatures leading to increased glacial meltwater and subsequently augmenting the inflow volume of the lake. Following temperature, precipitation had a significant influence, positively correlating with lake water level and area but with a 3-month lag. This pattern corresponds with research on other lakes in arid regions, such as Lake Chad, where the lake’s response to changes in watershed rainfall was delayed by 112 days (Gbetkom et al., 2023). The delay in lake water level and area changes compared to precipitation can be attributed to the arid soil’s high infiltration rates, which initially absorb increased precipitation and become saturated. As precipitation continues, reduced infiltration rates cause more runoff, gradually diminishing soil moisture input and causing the lag in lake changes (Wang et al., 2019b).

Furthermore, there was a negative correlation between lake water level, area, and the 3-month cumulative NDVI, potentially due to vegetation intercepting surface runoff, thereby reducing the volume of water entering the lake (Huang et al., 2023).

At the interannual scale (T > 24 months), which represents the overall trend, the correlations among lake water level, area, temperature, precipitation, and NDVI were weaker than those at the annual scale, and the lagged cumulative effects varied (**Supplementary Table S3**). As the temporal scale increased, the effects of temperature and NDVI on lake water levels accelerated gradually, whereas the influence of precipitation slowed down. Over longer timescales, gradual temperature changes and shifts in vegetation cover can have cumulative effects on lake water levels. For instance, increasing temperatures may enhance evaporation rates, contributing to a more pronounced effect on water levels over time (Woolway et al., 2020; Zhai and Tao, 2021). Similarly, changes in vegetation cover can affect runoff patterns and water retention, resulting in gradual and amplified effects on lake water levels (Zhai and Tao, 2021). Precipitation, particularly in arid and semiarid regions, can exhibit high annual variability. Over longer time scales, the influence of individual precipitation events might average out, leading to a reduced impact on lake water levels compared to other factors like temperature and vegetation changes. Research has indicated that since the mid-1980s, under the influence of global climate change, extreme precipitation events in the arid northwestern region of China have shown trends of increased intensity, duration, and frequency (Hu et al., 2021). The Tianshan region in Xinjiang stands out for its frequent extreme hydrological events, marked by the highest annual extreme precipitation occurrences, particularly concentrated in the central Tianshan mountain area with notable intensity (Yao et al., 2018). This increase in both the frequency and intensity of extreme precipitation events has led to increased annual precipitation, significantly affecting the interannual variability of the Kaidu River’s runoff. Additionally, the arid nature of the region’s soil could lead to high initial infiltration rates (Wang et al., 2019b), causing increased precipitation to be absorbed and not immediately reflected in the lake water levels. Therefore, these varying characteristics might result in longer-lasting influences of temperature and NDVI on lake water levels over extended time scales, whereas the effects of precipitation may be more pronounced in the short term but exhibit greater fluctuations over time.

### 4.4 Temporal variability in vegetation response to climate and its possible reasons

**Figure 14:**
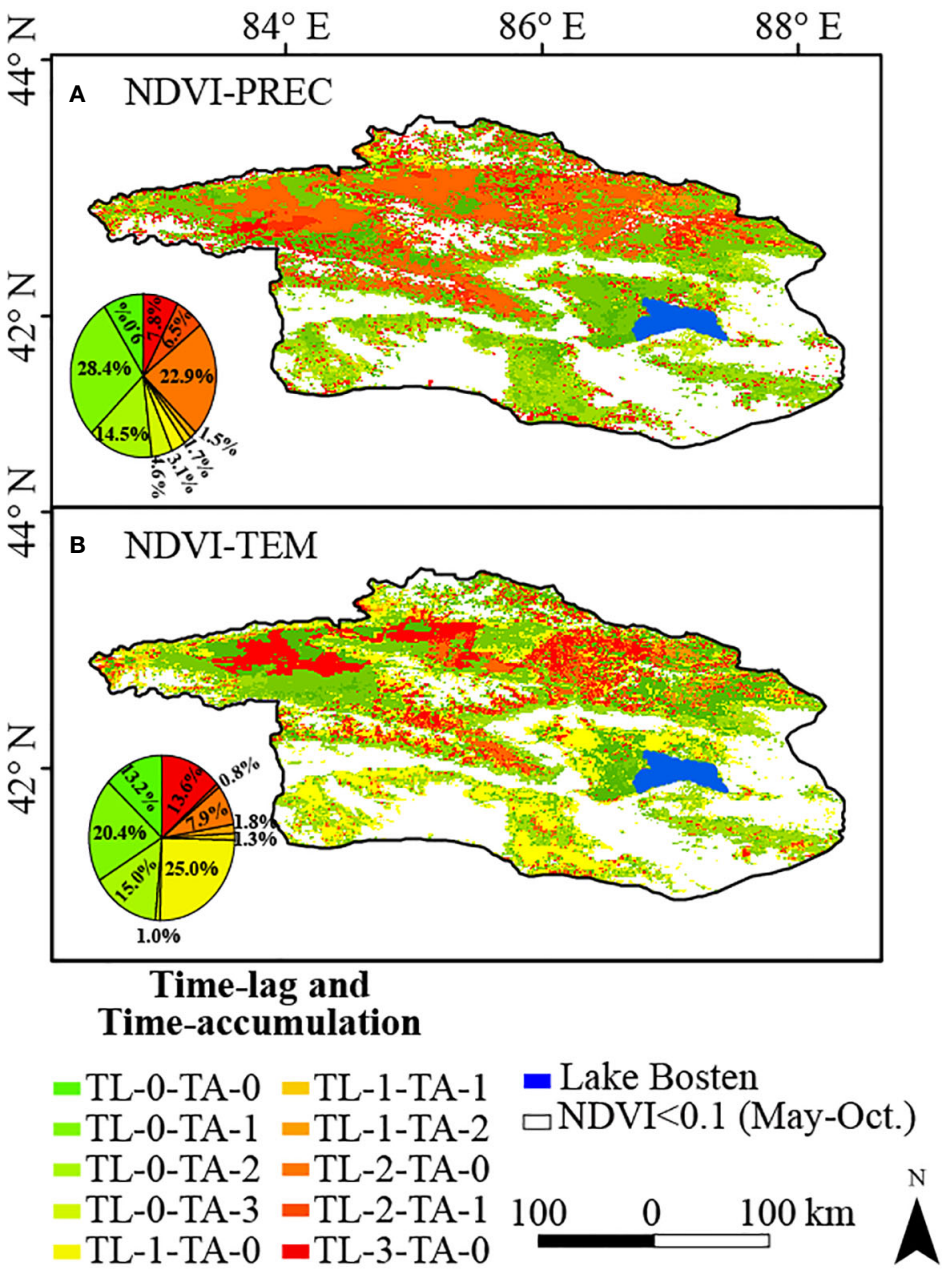
The effects of **(A)** precipitation, **(B)** temperature time-lag and time-accumulation to NDVI in the Bosten Lake Watershed from 2001 to 2020.

We also calculated the spatial pattern of the time-lag and time-accumulation months of climatic factors affecting NDVI on a monthly scale in the Bosten Lake watershed from 2001 to 2020 (**Figure 14**). The main combinations of time-lag and time-accumulation months for precipitation were TL-0-TA-1 (0-month lag and 1-month accumulation), TL-2-TA-0, and TL-0-TA-2, accounting for 28.4%, 22.9%, and 14.5%, respectively (**Figure 14**). The primary combinations for temperature were TL-1-TA-0 (1-month lag and 0-month accumulation), TL-0-TA-1, and TL-3-TA-0, accounting for 25.0%, 20.4%, and 13.6%, respectively (**Figure 14**). Generally, the time-lag effects of precipitation and temperature were significant in the western and northern parts of the study area, whereas the time-accumulation effects were significant around Bosten Lake. However, in the southern part of the study area, the time-accumulation effect of precipitation and the time-lag effect of temperature played a role simultaneously.

The climatic and topographical conditions in the northwestern part of the watershed likely resulted in a complex interplay among precipitation, temperature, and vegetation growth. Glacial meltwater serves as a crucial water source for vegetation growth in this region (Yao et al., 2018). Rising temperatures lead to increased glacier melting, higher runoff, enhanced vegetation coverage, and ecological benefits (Li et al., 2015). The intricate interactions of precipitation with soil cycling and plant transport gradually supply the needed water through soil infiltration and root absorption (Bodner et al., 2015). Due to the delayed effects of this process, the response of vegetation growth to precipitation and temperature may exhibit a lag. In contrast, in the southern region, with lower elevation and relatively sparse vegetation, precipitation can be swiftly absorbed by the soil or discharged through surface runoff. However, the cumulative impact of precipitation may accumulate over time, augmenting soil moisture content and stimulating vegetation growth. Conversely, temperature variations might lag in impacting vegetation growth, with limited vegetation cover leading to a more direct influence of temperature on soil and water heat exchange processes (Bodner et al., 2015).

This study explored the multi-timescale relationships between hydrology, vegetation dynamics and climate change in the Bosten Lake basin. It provided a scientific understanding of water resource management (lake chief scheme) in the basin to help policy formulation. With better observational data (high spatial and temporal resolution), the methodology of this study can be applied to better understand long-term trends and patterns. The interactions between climatic factors, hydrology, vegetation and human activities are complex, and the long-term impacts of climate change on ecosystems remain uncertain. Therefore, future research will require the development of predictive models and scenario simulations combining hydrological, ecological and socio-economic data.

### 4.5 Watersheds and SDGs in dryland: a comparative analysis

This study examined the relationship between lakes, vegetation, and climate change in dryland watersheds, which was relevant to the advancement of the SDGs. Climatic factors are the dominant factors of lake and NDVI changes in dryland watersheds (Woolway et al., 2020). By comparison, most studies indicated that temperature exerted a more significant impact on vegetation (Tan et al., 2018; Zhang et al., 2020; Rousta et al., 2023). However, in both the Aral Sea study and this study, it was found that the impact of precipitation on vegetation was more significant (Duan et al., 2022). The amount of precipitation determines the amount of surface water available (Berdimbetov et al., 2021). Vegetation interception plays a moderating role in watersheds of drylands (Zhang et al., 2022a). Ecological restoration therefore focuses on restoring vegetation and enhancing its resistance. Compared to previous studies, this study explored the lag effect of climate on NDVI in watershed landscapes, which aided in the development of ecological policies for arid ecosystems (Dai et al., 2020). In addition, the studies found that warmer temperatures and increased evapotranspiration were the primary drivers of lake shrinkage in drylands (Kiage and Douglas, 2020; Tuersun et al., 2020). It is noteworthy that the periodicity between them is more vital among climate, NDVI, and lake changes. Human activities can have positive effects after recognizing ecological and hydrological processes. Anthropogenic water resource management has been effective in mitigating the decline in ecosystem functioning within the watershed caused by climate change (Bryan et al., 2018). We recommend researchers should clarify not only the interrelationships between climate, vegetation, and lakes but also time-lag and time-accumulation. These findings can help in developing more resilient ecosystem management strategies by considering ecological and hydrological processes to advance the SDGs for dryland ecosystems.

## 5 Conclusion

Bosten Lake, China’s largest inland freshwater lake, has undergone significant transformations in recent years. This study employed the EEMD method to analyze the multi-time scale relationship among surface water, vegetation dynamics, and climate change in the Bosten Lake watershed. The water level and area of Bosten Lake have experienced three distinct phases: rapid decline (January 2001–June 2006), stability (June 2006-January 2016), and gradual increase (January 2016-December 2020). Vegetation within the watershed exhibited notable fluctuations, with 7.7% of the vegetation demonstrating a greening trend. Human activities have influenced the original time series, resulting in limited correlation at the monthly scale. Significantly, EEMD uncovered the underlying trends and correlations in the data. The correlations at the intra-annual scale surpassed those at the interannual scale. With an increasing time scale, the cumulative lag effect becomes more pronounced, accelerating the influence of temperature and NDVI values on lake water levels, while diminishing the impact of precipitation. The outcomes of this study enhance our understanding of the complex relationships among surface water, vegetation dynamics, and climate change across multiple timescales.

## Data availability statement

Publicly available datasets were analyzed in this study. This data can be found here: https://hydroweb.theia-land.fr/, http://www.geodata.cn.

## Author contributions

XG: Formal analysis, Funding acquisition, Resources, Writing – original draft, Writing – review & editing. JD: Data curation, Project administration, Supervision, Validation, Writing – review & editing. NA: Data curation, Investigation, Methodology, Software, Writing – review & editing. JX: Formal analysis, Visualization, Writing – review & editing. JW: Investigation, Resources, Supervision, Writing – original draft.

## Supplementary material

Supplementary Table 1 Time-Lag and time-accumulation correlation analysis combination.

Supplementary Table 2 Correlation coefficient statistics of raw data.

Supplementary Table 3 Results of partial correlation and time-lag (-accumulation) analysis.

Supplementary Figure 1 Applying EEMD to extract seasonal, annual, and interannual components of Areal time series in the Bosten Lake Watershed. “T” means the average period of each IMF.

Supplementary Figure 2 Applying EEMD to extract Cseasonal, Cannual, and Cinterannual components of Water level.

Supplementary Figure 3 Applying EEMD to extract Cseasonal, Cannual, and Cinterannual components of NDVI.

Supplementary Figure 4 Applying EEMD to extract Cseasonal, Cannual, and Cinterannual components of precipitation.

Supplementary Figure 5 Applying EEMD to extract Cseasonal, Cannual, and Cinterannual components of temperature.
