# Supplementary figures and images for: Responses of vegetation cover to hydro-climatic variations in Bosten Lake Watershed, NW China
Source: Front Plant Sci. 2024 Apr 16;15:1323445. doi: 10.3389/fpls.2024.1323445 (PMC11058830; doi:10.3389/fpls.2024.1323445)

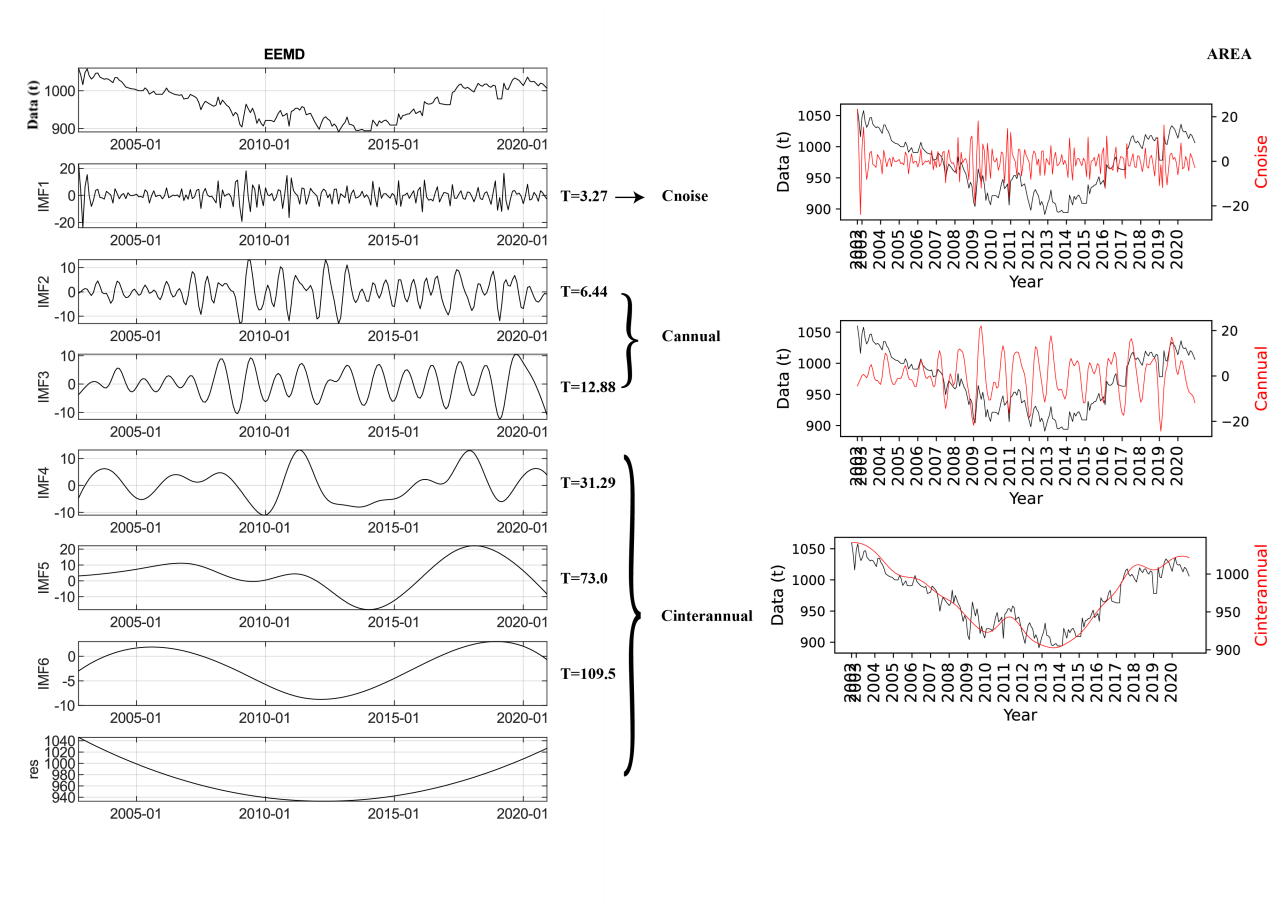

Supplement: Supplementary Figure 1 — Applying EEMD to extract seasonal, annual, and interannual components of Areal time series in the Bosten Lake Watershed. “T” means the average period of each IMF. [file Image_1.png]

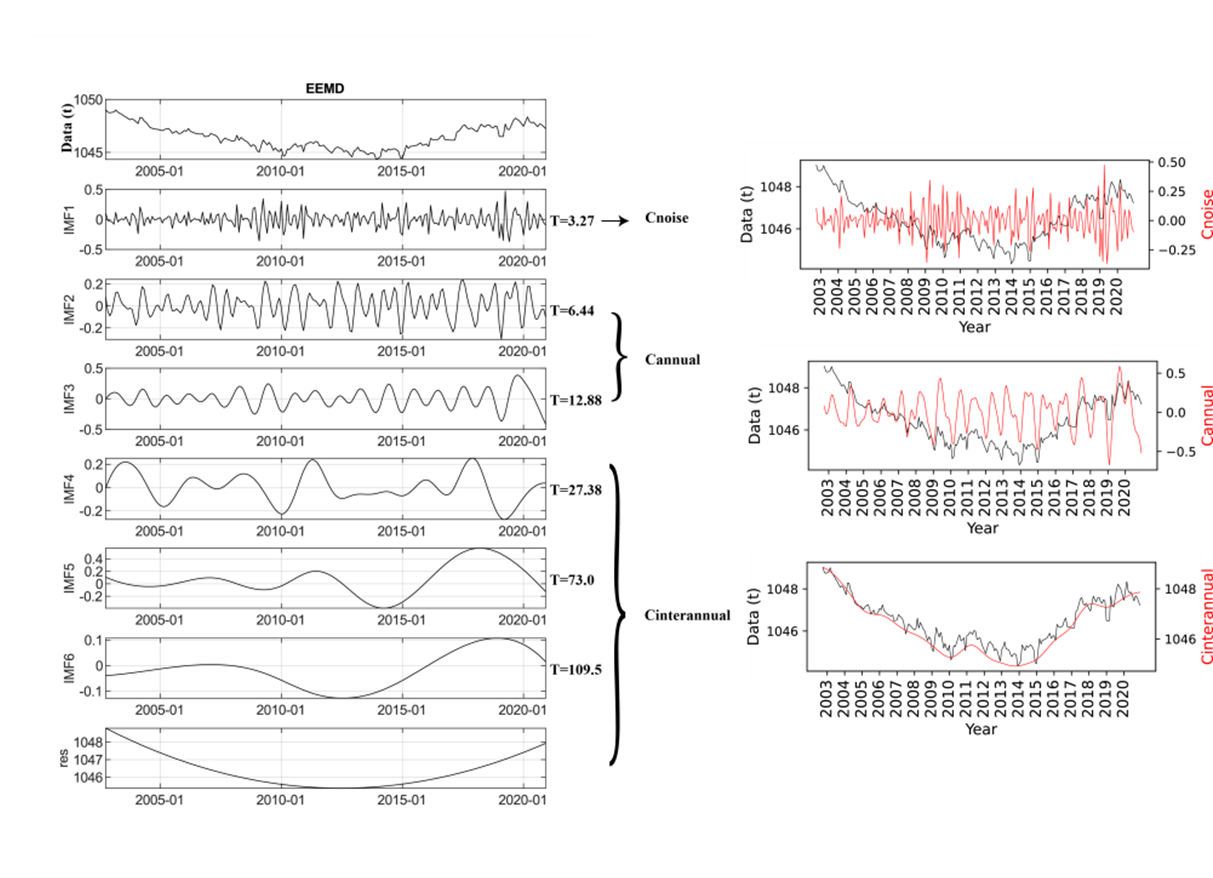

Supplement: Supplementary Figure 2 — Applying EEMD to extract Cseasonal, Cannual, and Cinterannual components of Water level. [file Image_2.png]

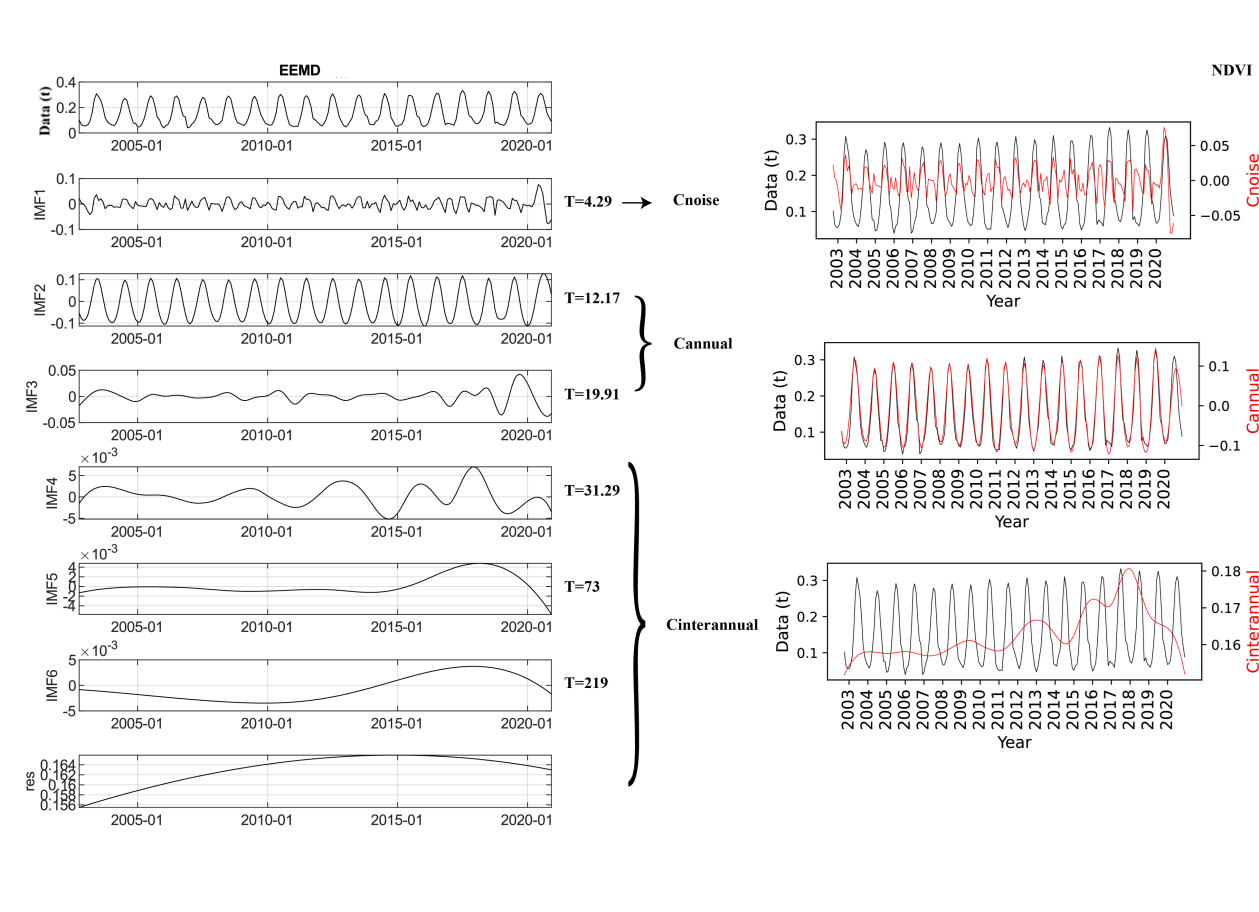

Supplement: Supplementary Figure 3 — Applying EEMD to extract Cseasonal, Cannual, and Cinterannual components of NDVI. [file Image_3.png]

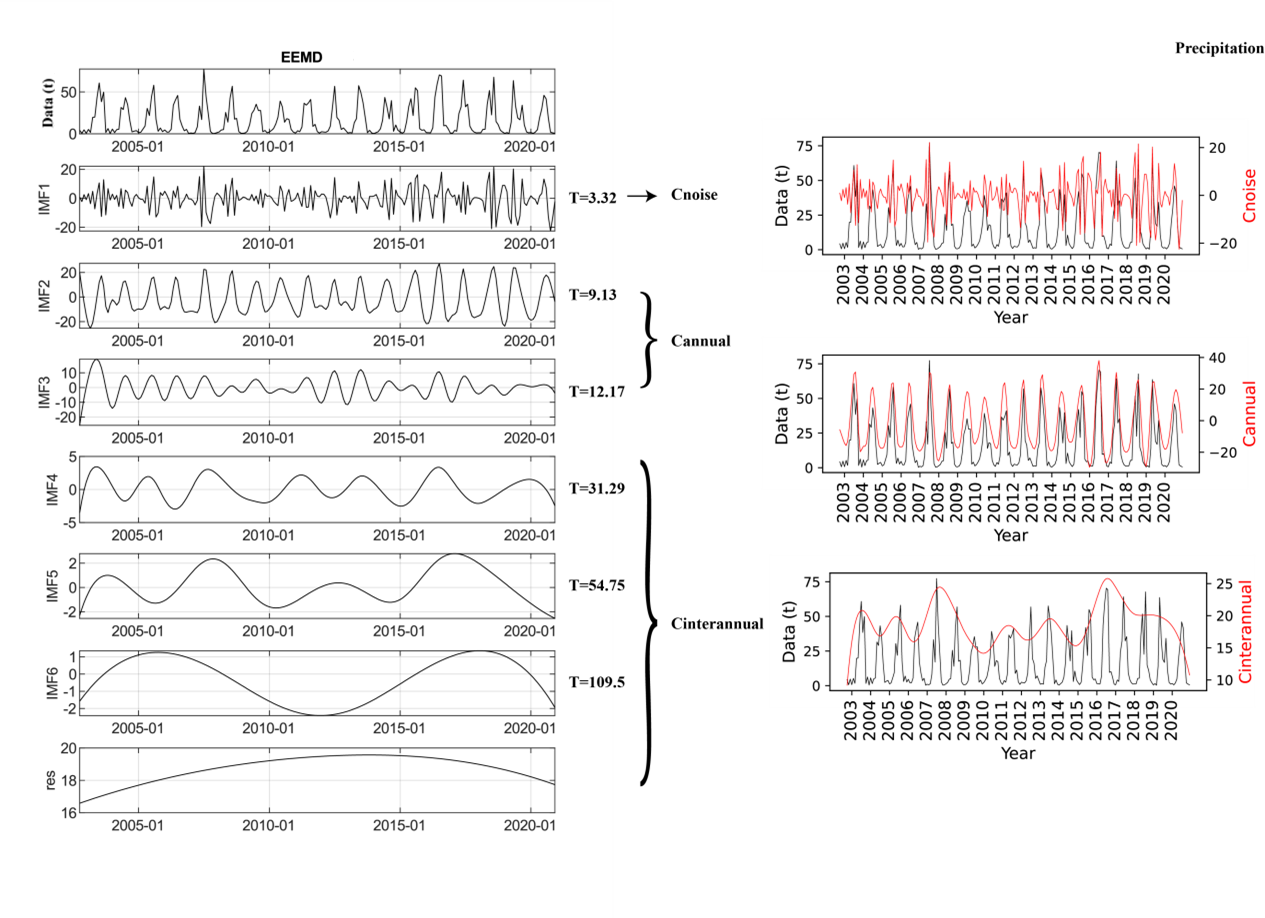

Supplement: Supplementary Figure 4 — Applying EEMD to extract Cseasonal, Cannual, and Cinterannual components of precipitation. [file Image_4.png]

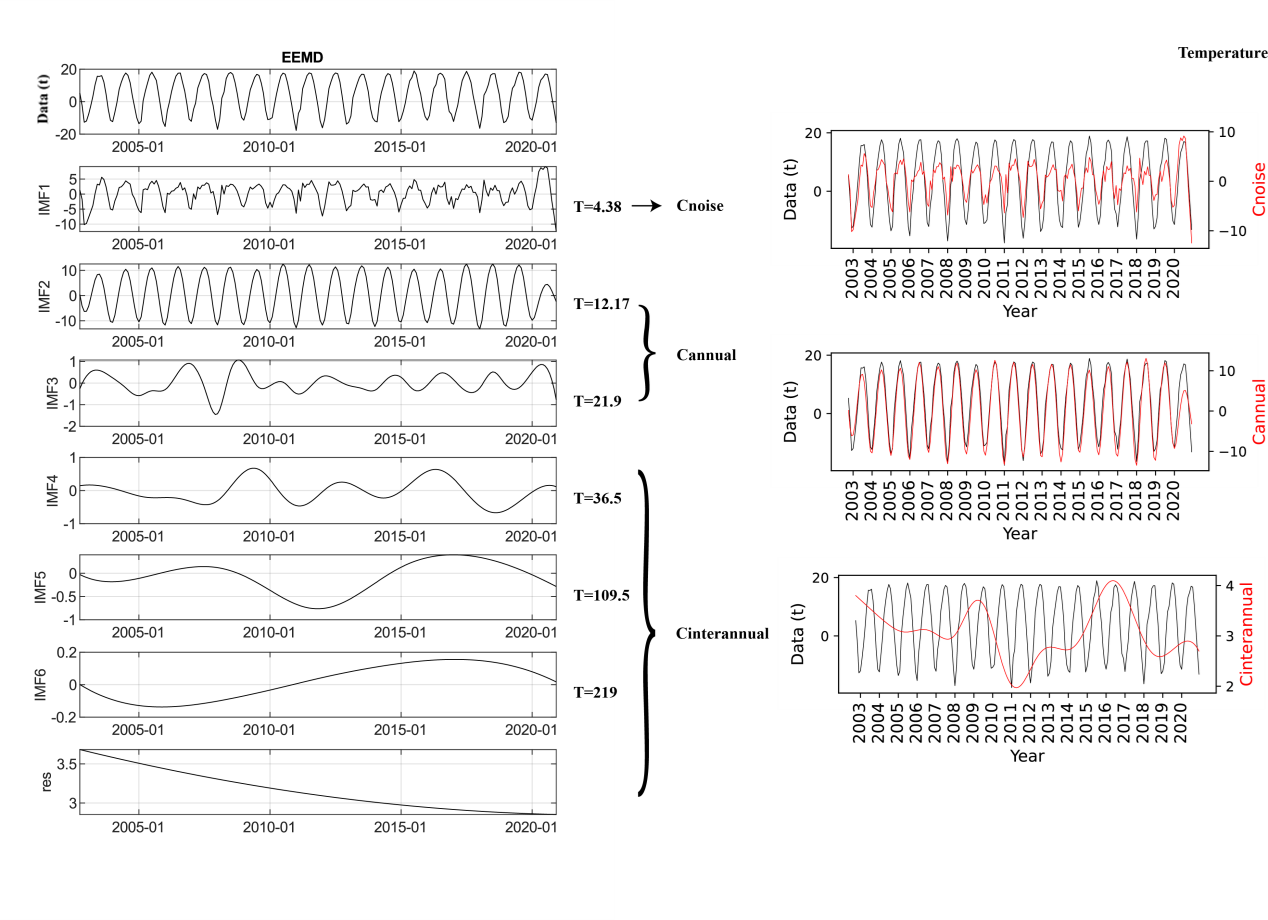

Supplement: Supplementary Figure 5 — Applying EEMD to extract Cseasonal, Cannual, and Cinterannual components of temperature. [file Image_5.png]
